# Cosmogenic nuclide dating of *Australopithecus* at Sterkfontein, South Africa

**DOI:** 10.1073/pnas.2123516119

**Published:** 2022-06-27

**Authors:** Darryl E. Granger, Dominic Stratford, Laurent Bruxelles, Ryan J. Gibbon, Ronald J. Clarke, Kathleen Kuman

**Affiliations:** ^a^Department of Earth, Atmospheric, and Planetary Sciences, Purdue University, West Lafayette, IN 47907;; ^b^School of Geography, Archaeology and Environmental Studies, University of the Witwatersrand, Johannesburg, WITS 2050, South Africa;; ^c^TRACES (Travaux et Recherches Archéologiques sur les Cultures, les Espaces et les Sociétés), UMR 5608 of the CNRS, Jean Jaurès University, Toulouse, 31058, France;; ^d^Private address, Cape Town 7800, South Africa;; ^e^Evolutionary Studies Institute, University of the Witwatersrand, Johannesburg, WITS 2050, South Africa

**Keywords:** *Australopithecus*, Sterkfontein, cosmogenic, burial, karst

## Abstract

Sterkfontein is the most prolific single source of *Australopithecus* fossils, the vast majority of which were recovered from Member 4, a cave breccia now exposed by erosion and weathering at the landscape surface. A few other *Australopithecus* fossils, including the StW 573 skeleton, come from subterranean deposits [T. C. Partridge *et al.*, *Science* 300, 607–612 (2003); R. J. Clarke, K. Kuman, *J. Hum. Evol.* 134, 102634 (2019)]. Here, we report a cosmogenic nuclide isochron burial date of 3.41 ± 0.11 million years (My) within the lower middle part of Member 4, and simple burial dates of 3.49 ± 0.19 My in the upper middle part of Member 4 and 3.61 ± 0.09 My in Jacovec Cavern. Together with a previously published isochron burial date of 3.67 ± 0.16 My for StW 573 [D. E. Granger *et al.*, *Nature* 522, 85–88 (2015)], these results place nearly the entire *Australopithecus* assemblage at Sterkfontein in the mid-Pliocene, contemporaneous with *Australopithecus afarensis* in East Africa. Our ages for the fossil-bearing breccia in Member 4 are considerably older than the previous ages of ca. 2.1 to 2.6 My interpreted from flowstones associated with the same deposit. We show that these previously dated flowstones are stratigraphically intrusive within Member 4 and that they therefore underestimate the true age of the fossils.

## Significance

*Australopithecus* fossils from the richest hominin-bearing deposit (Member 4) at Sterkfontein in South Africa are considerably older than previously argued by some and are contemporary with *Australopithecus afarensis* in East Africa. Our dates demonstrate the limitations of the widely accepted concept that *Australopithecus africanus*, which is well represented at Sterkfontein, descended from *A. afarensis*. The contemporaneity of the two species now suggests that a more complex family tree prevailed early in the human evolutionary process. The dates highlight the limitations of faunal age estimates previously relied upon for the South African sites. They further demonstrate the importance of detailed stratigraphic analysis in assessments of accurate dating of the karst cave sites in South Africa, which are stratigraphically highly complex.

The taxonomy, phylogeny, and chronology of *Australopithecus* in South Africa have long been controversial, with the site of Sterkfontein central to the debate (1–8). Fossils at the sites of Sterkfontein and Makapansgat in the Cradle of Humankind have been generally classed as *Australopithecus africanus* (9), but both assemblages have been recognized to include a second species (10), *Australopithecus prometheus* (11), with some cranial and postcanine dental morphology similar to *Paranthropus*, which suggested it might have been ancestral to that genus. A previous cosmogenic isochron burial date of 3.67 ± 0.16 million years (My) (2) places the *A. prometheus* skeleton StW 573 from the Silberberg Grotto, which is low within the Sterkfontein Formation (12), similar in age to *Australopithecus afarensis* at Laetoli (13) and late *Australopithecus anamensis* at Woranso-Mille (14). Previous burial dating in Jacovec Cavern, a separate chamber low within the Sterkfontein cave system, showed that *Australopithecus* fossils there are similar in age to StW 573 (1). These ages have been challenged, however, because they are much older than estimates for the *Australopithecus*-bearing breccia from higher in the cave (3–5). Here, we provide burial dates for these higher *Australopithecus*-bearing breccias. We also provide stratigraphic evidence to reconcile the relatively old ages determined from cosmogenic nuclide dates of the breccia with much younger ages determined from dating flowstones within the breccia using U-Pb and paleomagnetic dating (4, 5) at Sterkfontein.

**Fig. 1.**
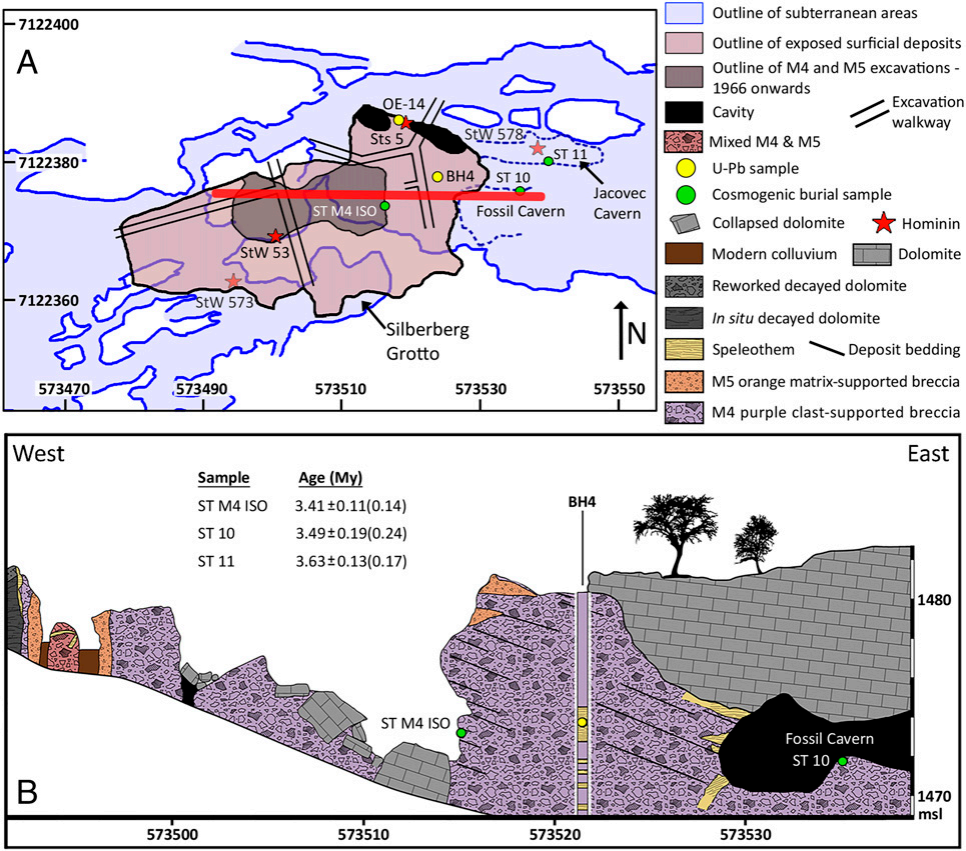
Map and cross section of Sterkfontein showing sample locations. (*A*) Map shows the extent of surface deposits and excavations superposed on the cave system. Sample locations reported here are shown as green circles; selected hominin fossils are shown with red stars and U-Pb-dated samples with yellow circles. Universal Transverse Mercator (UTM) coordinates are shown. (*B*) Cross section of the surface deposits along east-west red line in *A*. Cosmogenic sample locations are in green circles, and flowstone sample BH4-9 from ref. 5 in BH 4 is shown as a yellow circle. Measured bedding shows that the flowstone is located stratigraphically between the cosmogenic samples, although like other flowstones in Member 4, it is likely intrusive and younger than the breccia. Cross-section topography based on light detection and ranging (LiDAR) collected at the surface and underground. Borehole 4 stratigraphy is based on ref. 5.

The main body of the Sterkfontein cave fills has been divided into six members (12), with Members 1 to 3 underground and Members 4 to 6 now exposed through erosion of the cave roof (12; Fig. 1). The bulk of the *Australopithecus* fossil assemblage was recovered from Member 4, excepting the skeleton StW 573 from Member 2 (1, 2, 11) and a small assemblage from Jacovec Cavern (1). The StW 53 cranium was assigned to a phase of infill distinct from Member 5 but of uncertain age (15), but it is now shown to be a remnant of Member 4 (16); the deposit’s fauna and age need further study because solution pockets and erosion have significantly affected the breccia. Faunal correlations with sites in East Africa generally indicate a Late Pliocene or Early Pleistocene age for Member 4 (6), although localized mixing between Member 4 and the overlying Member 5 is very likely in parts of the site and the significant stratigraphic complexity at Sterkfontein was not recognized during most of the excavations. Stratigraphic records were not kept during excavations by P. V. Tobias and A. R. Hughes from 1976, and the presence of the younger member above the Type Site where fossils were blasted out and studied by R. Broom in the 1930s and Broom and J. T. Robinson (1947 to 1949) was not recognized at that time. Electron spin resonance (ESR) dating of fossil teeth exhibits a large spread from ∼1 to 4 My (7, 8), suggesting complex uranium uptake or mixing. Due to the potential for open system behavior and evidence for later fluid flow and carbonate deposition throughout the Member 4 breccia, we consider the ESR ages unreliable.

**Fig. 2.**
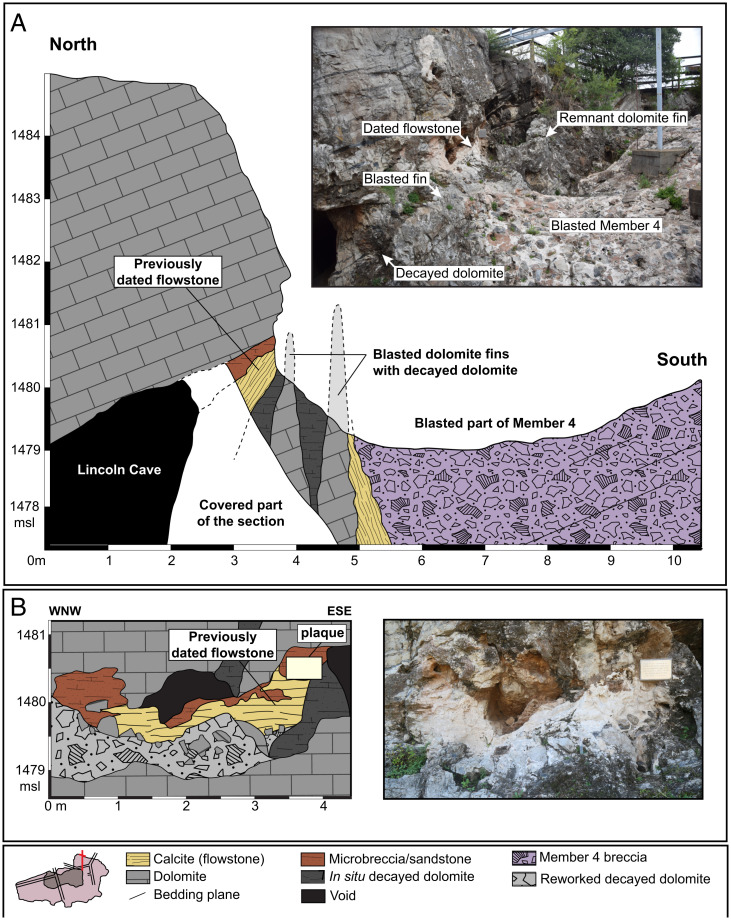
Stratigraphic sections and associated photos showing previously dated flowstone. Two sections are located at red bars shown in the base map found in the figure legend. (*A*) North-south section shows that the previously dated flowstone OE-14 (5) is not in stratigraphic contact with Member 4 but instead is separated by fins of dolomite and decayed dolomite that were removed by blasting. Its age therefore does not constrain that of Member 4. (*B*) Detailed section of the OE-14 flowstone (5) shows that it lies on decayed dolomite and reworked decayed dolomite breccia derived internally within the cave. The flowstone is overlain by and interfingers with orange sandy microbreccia with no clear stratigraphic relation to Member 4 or Member 5. The north-south cross section intersects at ca. 3.5 m on the west-northwest–east-southeast section, at the plaque.

Previous radiometric dating of Member 4 has been limited to U-Pb dates of flowstone. One such flowstone in the vicinity of the discovery site of the Sts 5 cranium (OE-14 of ref. 4; Figs. 1 and 2) dates to 2.03 ± 0.06 (2σ) My. A second flowstone (BH4-9 of ref. 4), recovered from a core taken in the eastern area of the exposed M4 breccia body (borehole 4; BH4 in Fig. 1), yields an age of 2.65 ± 0.18 (2σ) My. These two flowstones have previously been considered to bracket the top and bottom of Member 4 (4, 5); when combined with magnetostratigraphy of flowstone and adjacent fine-grained deposits, they place Member 4 from 2.07 to 2.61 My (5), which is much younger than the ∼3.7-My cosmogenic age (2) for Member 2, and approaching or overlapping *Paranthropus* and *Homo* at nearby Drimolen (17), Swartkrans (18, 19), and Sterkfontein Member 5 (2) and *Australopithecus sediba* at Malapa (20). However, there are three main problems with this interpretation for the age of the breccia, as follows:

1) The presumed top flowstone OE-14 does not directly constrain the age of Member 4. It grew in a cavity adjacent to the cave wall and was deposited directly upon autochthonous dolomite breccia and decayed dolomite (Fig. 2). It is separated from Member 4 by a vertical fin of dolomite that was removed by blasting but is still present along strike (Fig. 2). A fine-grained, well-bedded sandy breccia interstratifies the flowstone in its upper part, but there are no diagnostic features to correlate this sandy deposit with either Member 4 or the overlying Member 5. Instead, it is more likely to be derived from a separate entrance and deposited in a small cavity near the cave roof. There is no stratigraphic evidence that this flowstone was emplaced in sequence capping Member 4.
2) The presumed bottom flowstone BH4-9 actually lies in the upper middle part of Member 4 rather than at its base as previously supposed (Fig. 1). This is because the inferred dip of the talus cone in ref. 4 is incorrect due to a misconception that the bouldery talus facies in Member 4 is proximal rather than distal, requiring a talus cone dipping gently southwest. As discussed below, field observations (21) indicate instead a steep dip to the northeast (*SI Appendix*, Fig. S1), which places the flowstone at a higher stratigraphic level.
3) It is likely that most or all of the flowstones in the cores are intrusive, filling dissolved postdepositional cavities, as was previously demonstrated for Member 2 (22, 23) and is common in Member 4.

Because the interpretation of the dating of Member 4 relies so heavily on the cave infill stratigraphy, we describe the depositional setting here before presenting our results.

Member 4 accumulated as a talus cone within the cave, beneath a vertical entrance shaft. Cave talus cones exhibit many similarities with surface rockfall deposits (22, 23). In unconsolidated clast-rich deposits, the slope angle typically ranges between 28° and 38° (24–27). Larger and rounder rocks are transported to the flanks and toe of the cone, while smaller rocks tend to pile near the apex or become lodged in crevices (25, 26), leading to grain size segregation. In the talus proximal and medial slope, elongated clasts tend to align their long axes parallel to the talus cone bedding and glide downslope, suspended by the matrix (27). As a result, the proximal facies tends to be finer and matrix supported with bedding planes expressed in the fabric, while the distal facies tends to be more bouldery, clast supported, and open in structure (*SI Appendix*, Fig. S2). The edge of the talus cone typically grades into low-gradient, fine-grained sediments blanketing the cave floor (*SI Appendix*, Fig. S2). Relatively low-density vegetation that falls into the cave entrance commonly accumulates near the talus cone apex.

The Member 4 breccia has long been recognized as an exhumed talus cone due to its steeply dipping bedding that radiates from the southern part of the surface exposures (12, 28–31). Our survey of >1,000 elongated clast orientations within the breccia confirms a dip of 42° ± 16° down to the northeast (*SI Appendix, Text* and Fig. S1). The presence of fossilized liana in Member 4 (32), preferentially located near the southern end of the exposed talus (15, 33), provides additional evidence for a former entrance in the south. After sedimentation of Member 4 ceased, likely due to filling of the cave and choking of the entrance, the breccia was cemented by calcite and then partially dissolved and eroded into an irregular surface including cavities within the breccia. Member 5 then entered from a separate entrance further to the east and blanketed Member 4 unconformably (Fig. 1 and *SI Appendix*, Fig. S1), intruding into some of the dissolved cavities.

Many of the cavities that formed within Member 4 subsequent to its deposition were filled with calcite flowstone. In 1947, Haughton noted within the breccia “prominent almost horizontal but irregularly thick veins of [white calcite] which have, undoubtedly, been formed subsequent to the deposition of the breccia” (34). More recent stratigraphic analyses (2, 22, 23) showed clear evidence of intrusive flowstone formation in Member 2 in the Silberberg Grotto. Here, we document similar relationships in Member 4, in a section exposed at the western end of Fossil Cavern, down-dip of the BH4-9 sample (*SI Appendix*, Fig. S3). These flowstones exposed in the Fossil Cavern are without exception intrusive and younger than the breccia in which they are found, even though they can lie parallel to bedding. Evidence for their intrusive nature comes from solutional unconformable contacts, as well as blocks derived from the cemented breccia that are embedded in the calcite flowstone (*SI Appendix*, Fig. S3).

The sedimentary fabric and architecture indicate strongly that Member 4 accumulated as a talus cone radiating from the southern edge of the exposed breccia (12, 15, 28–31). The sedimentary facies provide additional evidence for this interpretation. A finer-grained, matrix-supported facies with plant fossils (33) proximal to the entrance transitions to a bouldery, clast-supported, matrix-poor facies distally, typical of accumulation at the bottom of a shaft (22). However, a considerably different model has been presented in the literature based on interpolation among five widely separated sediment cores distributed around the periphery of the exposed breccias (*SI Appendix*, Fig. S4; 4, 35). The cores were correlated based on the transition from autochthonous to allochthonous material, indicating the base of the externally derived talus, as well as on the presence of flowstone layers, which were assumed to be synchronous if not continuous across the boreholes, and deposited in ascending sequence with the breccia during deposition. Several of these flowstones were dated with U-Pb and correlated across the cores (4). The sources of the breccia were then interpreted based on a longitudinal facies attribution in which coarse bouldery breccia with scant matrix was considered proximal to the cave entrance, a finer-grained matrix-supported facies medial on the talus cone, and a fine-grained, horizontally bedded facies most distal (4). The cave entrances were interpreted to be associated with the boulder facies (4), implying that Member 4 formed a gently dipping surface emanating from an entrance to the northeast. These interpretations of the proximal and medial facies are opposite of expectations on a talus cone, and a cave entrance to the northeast is diametrically opposed to the observed steep talus dip from the southwest. Moreover, the flowstones upon which the stratigraphic correlations are based (4) are most likely intrusive and younger than the breccia in which they are found and cannot be correlated across the widely separated cores. We believe that the original field-evidence-based interpretation of a talus cone emanating from the south (12, 15, 28–31) is correct.

The recognition of serious stratigraphic problems with the interpretation of previous dating based on flowstones brings the true age of the Member 4 breccia and its fossils into question. Does *Australopithecus* in Member 4 date closer to the ∼3.7-My age of Member 2, or to the ∼2.1-My age of *Paranthropus*, *Homo*, and *A. sediba* (17–20)?

To date the Member 4 breccia directly, we use isochron burial dating with ^26^Al and ^10^Be on a suite of clasts collected from the deepest exposures in the excavation site (Fig. 1, sample ST M4 ISO). We also date a single sample of sandy matrix from the upper middle part of Member 4, collected from Fossil Cavern (Fig. 1, sample ST 10; “lower cave” of ref. 12), and a single sample of sand from Jacovec Cavern (Fig. 1, sample ST 11), supplementing previously reported data (1).

## Results

**Fig. 3.**
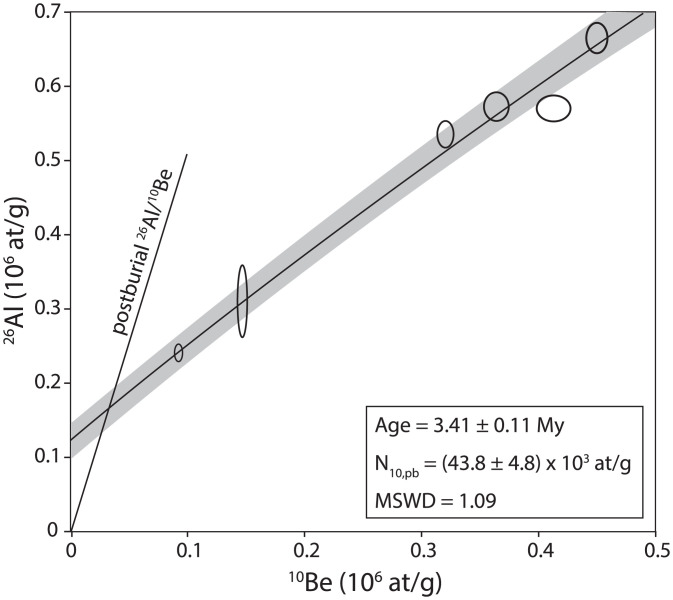
Member 4 isochron. Best-fit isochron curve with 1σ uncertainty band shaded. Postburial production is determined by the intercept of the isochron with the calculated postburial ^26^Al/^10^Be line. The isochron fits all data to within analytical uncertainty shown as 1σ error ellipses.

The isochron samples (ST M4 ISO; *SI Appendix*, Table S1) yield an age of 3.41 ± 0.11 My (1σ analytical error; Fig. 3), with a mean square weighted deviation of 1.09, indicating that all of the samples are consistent with a single burial age. The age estimate from the slope of the isochron is largely independent of the postburial production estimate from the intercept; however, the inferred postburial production provides an internal check of the assumptions in the model. The postburial ^10^Be production rate determined from the isochron fit is 0.028 ± 0.003 at g^−1^ yr^−1^, matching the expected value of 0.030 at g^−1^ yr^−1^at a depth of 10 m and density of 2.0 g cm^−3^ (36; *SI Appendix*, Table S2), lending confidence to the solution. In addition to analytical uncertainties, used when comparing cosmogenic nuclide ages to each other, systematic uncertainties in decay constants add 2% uncertainty to the final age, and a 3% uncertainty in the production rate ratio adds 0.05 My of uncertainty. Considered together, the best fit age is 3.41 ± 0.11(0.14) My, with the total uncertainty expressed in parentheses here and following. *Australopithecus* specimens are closely associated with the isochron samples, including one individual (StW 537) consisting of several mandibular teeth found less than a meter away (37). The partial skeleton StW 431 lies ∼2.5 m higher and 2 to 3 m northwest within the same deposit (38).

The sediment sample from Fossil Cavern (ST10; *SI Appendix*, Table S1) yields an age of 3.49 ± 0.19 (0.24) My (Table 1), which is indistinguishable within error from the isochron age, calculated using postburial production rates estimated for a depth of 11 m beneath a rock of density 2.8 g cm^−3^ (36; *SI Appendix*, Table S2). Four *Australopithecus* fossils were found here in 1937 (39), consisting of a maxilla (TM 1512), a distal femur (TM 1513), a crushed maxilla (TM 1514), and a capitate (TM 1526).

**Table 1.** Single-sample burial ages

Sample (location)	Burial age (My)
ST 10 (Fossil Cavern)	3.49 ± 0.19
ST 11 (Jacovec)	3.63 ± 0.13
ST 4* (Jacovec)	3.95 ± 0.21
ST 5* (Jacovec)	3.39 ± 0.21
Jacovec average	3.61 ± 0.09

*Previously reported in ref. 1, revised using ^10^Be standard (40) and half-life (57, 58).

Sample ST11 from Jacovec Cavern (*SI Appendix*, Table S1) yields a burial age of 3.63 ± 0.13(0.17) My (Table 1 and *SI Appendix*, Table S2), calculated using postburial production rates at a depth of 29 m in bedrock of density of 2.8 g cm^−3^ (36). This age agrees with two previously published dates (1), recalculated with the production rates used here and a revised ^10^Be standard and half-life (40; Table 1 and *SI Appendix*, Table S2). Together, the three Jacovec samples yield an average age of 3.61 ± 0.09(0.13) My. *Australopithecus* fossils recovered from Jacovec Cavern include cranium StW 578 and several postcranial fossils (1).

## Discussion

Our ages place the deposition of the bulk of Member 4 near 3.4 My, which is significantly older than the 2.61- to 2.07-My range previously proposed (5) but consistent with previous dating of lower deposits in the Silberberg Grotto and Jacovec Cavern. The Oldowan unit near the base of the overlying Member 5 was previously dated at 2.18 ± 0.21 (0.24) My using simple burial dating (2), implying a ∼1-My hiatus in deposition across the unconformity between Members 4 and 5. Such a long duration allows sufficient time to develop the unconformity, during which a large part of Member 4 was dissolved and eroded away, creating space to accommodate Member 5 after the opening of an entrance to the east.

Previous burial dating at Sterkfontein has been questioned because it is older than ages determined from U-Pb and paleomagnetic dating of flowstone and ESR dating of fossil teeth (3–5). Although burial dating of individual samples can be subject to error due to reworking (3), it is highly unlikely that an isochron burial date would be subject to the same problem unless all of the sediment was reworked from a higher unconsolidated deposit within the cave. There is no evidence of any older, higher level at Sterkfontein from which sediment could be remobilized; the presence of liana and vertebrate fossils strongly indicates that the cave was open to the surface at the time of deposition, and the stratigraphy indicates gradual accumulation beneath a steep entrance shaft to the south. The isochron burial dating method is based on well-understood physics of cosmogenic nuclide production and radioactive decay; its accuracy has been tested against ^40^Ar/^39^Ar dating of volcanic flows (41) and paleomagnetism (42) and is consistent with U-Pb dating of flowstone at Swartkrans (19). Only at Sterkfontein where there is demonstrable evidence of intrusive flowstone deposition is there a significant disagreement between isochron burial dating and other absolute dating methods. We believe that previous dating of flowstones by U-Pb and paleomagnetism is correct but that the dates indicate the age of flowstone deposition rather than accumulation of the breccia. The discrepancy between older burial dates and younger flowstone ages is entirely due to their stratigraphic relationship. The age of the fossils is best represented by the breccia in which they are encased.

Concerns about an older age for Member 4 have been raised based on faunal considerations as well (4, 5) However, faunal ages must be viewed cautiously because early work did not recognize the boundary between Members 4 and 5, and vertical mixing between the members can occur by subsidence and bioturbation in decalcified solution pockets that permeate the site (15, 30, 43; *SI Appendix*, Fig. S5). Because Hughes excavated mechanically in spits (3’ × 3’ [0.91 m x 0.91 m] in area and 1’ [0.30 m] deep) and did not observe stratigraphic detail, the only record we have of the numerous solution pockets in Member 4 is photographic (e.g., *SI Appendix*, Fig. S5). Sediments in solution pockets are typically stained dark by manganese released from the dolomite during weathering by humic acids.

*Equus*, a genus younger than ∼2.3 My in Africa and incompatible with our ages, has been assigned to Member 4 (44) based on material from the Broom collection (1936 to 1948). However, Broom excavated by blasting in an area that is now known to be overlain by the younger Member 5 (15, 30; Fig. 1 and *SI Appendix*, Fig. S2), at a time long before the younger member had been identified. Subsequent work (15) using only provenanced fossils from the later Hughes excavation noted a single *Equus* tooth from Member 4 (S94-323; *SI Appendix*, Fig. S6) recovered from square O/42 (spit level 16’4” to 17’4” [4.98 to 5.28 m]). This tooth shows manganese staining indicating that it derives from a solution pocket (*SI Appendix*, Fig. S6) and is likely intrusive from Member 5. Two additional postcranial fossils from the Hughes excavations within the Member 4 area were identified by Kibii (45) only to family level as *Equid*. One is a distal fragment of a radius (S94-13118-19), from square O/46 at 5’5” to 6.5” (1.65 to 1.96 m) below datum. This specimen could not be located for checking, but an analysis of closely associated lithics by K.K. shows manganese staining indicative of a solution pocket in Square O/46 at 12’2” (3.71 m), and thus, this specimen should also be considered intrusive. The second specimen (S94-11418), found deeper in Square U/47 at 17’3” to 18’3” (5.26 to 5.56 m) below datum, was identified as an equid magnum (45). However, R.J.C. has examined the bone and determined that it is instead the fused left magnum and unciform of a Class-3-sized bovid (*SI Appendix*, Fig. S7). Our dating now adds strong support to the argument that all *Equus* fossils have been incorrectly assigned to Member 4 (*SI Appendix*, *Text*).

Similar concerns arise regarding other young taxa assigned to Member 4, including the suid *Metridiochoerus* (Sts 3074; 46, 47) and the springbok *Antidorcas* (44), which are both limited to specimens from the 1936 to 1948 Broom collection (44). *Metridiochoerus* is known from Usno dating to 3.4 My but appears more primitive in its third molar than the South African examples (47). *Antidorcas* is present at Shungura B10 dating to 2.9 My (48). In summary, the poorly provenanced taxa discussed above demonstrate the complications that have been created and perpetuated in the literature for many years regarding faunal age estimates for Member 4. We are certain from the remaining stratigraphy preserved at the site that an eastward extension of Member 5 was present overlying Member 4 during Broom’s excavations (Fig. 1 and *SI Appendix*, Fig. S2; 15, 30). If even a small number of the fossils was incorrectly attributed to Member 4, we cannot reject a radiometric age based on the presence of these limited younger taxa.

Ecological reconstructions from the Member 4 fauna indicate a climate more humid than today, with mosaic grassland, savanna, and gallery forest, consistent with both C_3_ and C_4_ diets determined from fossil teeth (49). The Member 5 fauna is associated with a drying climate and far more grazers (49). It was previously assumed that the faunal transition from Member 4 to Member 5 occurred rapidly across the Pliocene to Pleistocene climate transition near 2.5 My (6, 50); however, our dates show that the more humid assemblage refers to the mid-Pliocene, prior to the mid-Piacenzian Warm Period, while the drier assemblage refers to the late Early Pleistocene, consistent with a trend toward decreasing weathering intensity over the same time period inferred from marine records in the Mozambique Channel (51). The Pliocene to Pleistocene climatic transition, thought to be important for the emergence of *Homo*, is not represented at Sterkfontein, although there was flowstone deposition in the cave at that time (4, 18).

Our dates show that the entire *Australopithecus* assemblage at Sterkfontein dates to ca. 3.4 to 3.7 My. These australopiths were thus early representatives of the genus, overlapping in age with a morphologically diverse range of mid-Pliocene hominins, including *A. afarensis* (13), *Australopithecus deyiremeda*, and the unassigned foot BRT-VP-2/73 at Burtele (52, 53), *Australopithecus bahrelgazali* in Chad (54), *Kenyanthropus platyops* at Lake Turkana (55), and late *A. anamensis* at Woranso-Mille (14). The Sterkfontein australopiths predate *Paranthropus*, *Homo*, and *A. sediba* at nearby sites in the Cradle of Humankind by over a million years, providing a fuller picture of hominin presence and evolution in southern Africa and increasing the geographic range and taxonomic diversity of hominins during the mid-Pliocene.

## Materials and Methods

### Cosmogenic Nuclide Burial Dating.

Isochron burial dating is based on the relative radioactive decay of ^26^Al (t_1/2_ = 0.708 ± 0.056 My; 56) and ^10^Be (t_1/2_ = 1.387 ± 0.012 My; 57, 58) in quartz clasts that were first exposed to secondary cosmic radiation near the ground surface and subsequently buried (59, 60). Each individual clast contains ^26^Al and ^10^Be that built up as it was eroded from bedrock and exposed at the surface, as well as any nuclides that accumulated after burial. If all of the clasts were buried simultaneously, they will have the same burial age and the same postburial production history. Assuming that samples are derived from a steadily eroding landscape and that postburial production has occurred at a constant rate, the relationship between ^26^Al and ^10^Be is described by Eq. **1**.

[1] $$N_{26}=\left(N_{10}–N_{10,pb}\right)[\left(P_{26}/P_{10}\right)exp(-t/\tau_{\mathrm{bur}})/(1+\left(N_{10}–N_{10,pb}\right)exp(t/\tau_{10})/(P_{10}\;\tau_{\mathrm{bur}})]+N_{26,pb},$$

where *N_i_* represents the measured concentration of nuclide *i*, *P_i_* represents the surface production rate of nuclide *i*, *t* is burial age, τ_i_ represents the radioactive meanlife (τ = t_1/2_/ln[2]), N_i,pb_ is the concentration of nuclide i accumulated after burial, and τ_bur_ is an effective meanlife calculated as τ_bur_ = (τ_26_^−1^ – τ_10_^−1^)^−1^. Solution of Eq. **1** requires additional knowledge of postburial production. We assume that production has remained steady since burial, so the postburial concentrations follow Eq. **2**.

[2] $$N_{i,pb}=P_{i,pb}\tau_{\text{i}}(1-\exp(-t/\tau_{\text{i}})).$$

We use *P_26_,_pb_*/*P_10,pb_* = 8.5, in accordance with production rate calculations at depths of 10 to 11 m beneath the surface (36). The exact values for *N_i,pb_* are determined by fitting the data and can be compared to theoretical expectations to help validate the assumptions in the model.

Eq. **1** shows that a plot of ^26^Al versus ^10^Be follows a gentle curve with a slope determined by the burial age, curvature determined by the erosion rates, and an intercept determined by postburial production. Samples that have been reworked from older deposits will lie below the isochron curve; samples significantly above the curve would indicate laboratory error or mixing of a younger clast (19).

Simple burial dating works similarly to isochron burial dating using Eq. **1**, but for a single sample, and postburial production must be explicitly accounted for by modeling the production of ^26^Al and ^10^Be at depth using Eq. **2** and production rates from ref. 36. Ignoring postburial production yields a minimum burial age except where postburial production is negligible due to deep burial.

Here, we use the isochron burial dating method for the main excavation site of Member 4 and the simple burial dating method at Fossil Cavern and Jacovec Cavern. For the isochron, 11 blocks of chert were collected from the exposed excavation wall in the lower part of Alun Hughes’ Member 4 excavation; 6 of them were selected for analysis based on their degree of weathering, choosing as wide a variety as possible to maximize variability in inherited cosmogenic nuclide concentrations (*SI Appendix*, Fig. S8). Samples were collected from within a vertical interval of ∼0.5 m at Square T43, depth 33′8″ (10.26 m) in the coordinate system of ref. 61, or Lo: Y: −73,545.0; X: 2,878,777.5; Z: 1,473.5 (m asl) in the global coordinate framework described in ref. 62. The burial depth beneath the pre-excavation surface is ∼10 m. For simple burial dating, a sample of sandy matrix material was collected from Fossil Cavern at Lo: Y: −73,565.5; X: 2,878,774.4; Z: 1472.0, with a shielding depth of ∼11 m. Finally, a sample of sandy matrix was collected from Jacovec Cavern, near samples previously reported in ref. 4, at a location of Lo: Y: −73569; X: 2878769; Z: 1454.3, with a shielding depth of 29 m.

The chert blocks were crushed, and the 0.25- to 0.5-mm fraction from all of the samples was purified by repeated daily leaching in hot agitated 5% HF/HNO_3_. The cleaned quartz was dissolved in 5:1 HF/HNO_3_ and spiked with a ^9^Be carrier solution prepared in house. An aliquot of each digested sample was taken for determination of Al by inductively coupled plasma optical emission spectrometry. The remaining solution was evaporated to dryness in H_2_SO_4_ and converted to chloride form, and contaminating elements were separated by selective precipitation at pH >14. After precipitation of Al and Be at pH 8, the target elements were separated by ion exchange in 0.4 M oxalic acid and then converted to oxide by flame. Both Al and Be oxides were mixed with niobium and analyzed by accelerator mass spectrometry (AMS) at PRIME Lab, Purdue University against standards reported in refs. 40, 56. The measured concentrations of ^26^Al and ^10^Be are given in *SI Appendix*, Table S1.

All burial ages were calculated assuming a surface ^26^Al/^10^Be production rate ratio of 6.8 and that samples experienced steady erosion prior to burial. Following ref. 19, we use a local surface ^10^Be production rate of 9.8 ± 1.6 at/g/yr, calculated as the average and SD of the time-varying production rate at the site over the past 2 My (63). Postburial production by muons for the Member 4 isochron is taken at a ^26^Al/^10^Be ratio of 8.5 (*SI Appendix*, Table S2) based on ref. 36.

## Supplementary Material

Supplementary File

## Data Availability

Dip data have been deposited in WIReDSpace (https://wiredspace.wits.ac.za/handle/10539/32863).

## References

[1] T. C. Partridge, D. E. Granger, M. W. Caffee, R. J. Clarke, Lower Pliocene hominid remains from Sterkfontein. Science 300, 607–612 (2003). 12714736 10.1126/science.1081651

[2] D. E. Granger , New cosmogenic burial ages for Sterkfontein member 2 Australopithecus and member 5 Oldowan. Nature 522, 85–88 (2015). 25830884 10.1038/nature14268

[3] J. D. Kramers, P. H. Dirks, The age of fossil StW573 (‘Little Foot’): An alternative interpretation of ^26^Al/^10^Be burial data. S. Afr. J. Sci. 113, 8 (2017).

[4] R. Pickering, J. D. Kramers, Re-appraisal of the stratigraphy and determination of new U-Pb dates for the Sterkfontein hominin site, South Africa. J. Hum. Evol. 59, 70–86 (2010). 20605190 10.1016/j.jhevol.2010.03.014

[5] R. Pickering, A. I. R. Herries, “A new multidisciplinary age of 2.61-2.07 Ma for the Sterkfontein Member 4 australopiths” in Hominin Postcranial Remains from Sterkfontein, South Africa, 1936-1995, B. Zipfel, B. G. Richmond, C. V. Ward, Eds. (Oxford University Press, 2020), pp. 21–30.

[6] E. S. Vrba, Some evidence of chronology and palaeoecology of Sterkfontein, Swartkrans, and Kromdraai from the fossil Bovidae. Nature 254, 301–304 (1975).

[7] B. A. Blackwell, Problems associated with reworked teeth in electron spin resonance (ESR) dating. Quat. Sci. Rev. 13, 651–660 (1994).

[8] D. Curnoe, “A contribution to the question of early Homo in southern Africa: Researches into dating, taxonomy and phylogeny reconstruction,” PhD thesis, Australian National University, Canberra, Australia (1999)

[9] J. T. Robinson, The genera and species of the Australopithecinae. Am. J. Phys. Anthropol. 12, 181–200 (1954). 13188956 10.1002/ajpa.1330120216

[10] R. J. Clarke, “A new *Australopithecus* cranium from Sterkfontein and its bearing on the ancestry of *Paranthropus*” in Evolutionary History of the “Robust” Australopithecines, F. Grine, Ed. (Aldine de Gruyter, New York, 1988), pp. 285–292.

[11] R. J. Clarke, K. Kuman, The skull of StW 573, a 3.67 Ma Australopithecus prometheus skeleton from Sterkfontein Caves, South Africa. J. Hum. Evol. 134, 102634 (2019). 31446970 10.1016/j.jhevol.2019.06.005

[12] T. C. Partridge, Re-appraisal of lithostratigraphy of Sterkfontein hominid site. Nature 275, 282–287 (1978).

[13] A. L. Deino, “^40^Ar/^39^Ar dating of Laetoli, Tanzania” in Paleontology and Geology of Laetoli: Human Evolution in Context, T. Harrison, Ed. (Springer, 2011), pp. 77–97.

[14] Y. Haile-Selassie, S. M. Melillo, A. Vazzana, S. Benazzi, T. M. Ryan, A 3.8-million-year-old hominin cranium from Woranso-Mille, Ethiopia. Nature 573, 214–219 (2019). 31462770 10.1038/s41586-019-1513-8

[15] K. Kuman, R. J. Clarke, Stratigraphy, artefact industries and hominid associations for Sterkfontein, member 5. J. Hum. Evol. 38, 827–847 (2000). 10835264 10.1006/jhev.1999.0392

[16] R. A. Couzens, “Spatial Modelling, formation and transformation of the Oldowan lithic artefact assemblages from Sterkfontein Caves, South Africa,” PhD thesis, University of the Witwatersrand, Johannesburg, South Africa (2021).

[17] A. I. R. Herries , Contemporaneity of *Australopithecus*, *Paranthropus*, and early *Homo erectus* in South Africa. Science 368, eaaw7293 (2020). 32241925 10.1126/science.aaw7293

[18] R. Pickering, J. D. Kramers, P. J. Hancox, D. J. deRuiter, J. D. Woodhead, Contemporary flowstone development links early hominin bearing cave deposits in South Africa. Earth Planet. Sci. Lett. 306, 23–32 (2011).

[19] K. Kuman , A new absolute date from Swartkrans Cave for the oldest occurrences of Paranthropus robustus and Oldowan stone tools in South Africa. J. Hum. Evol. 156, 103000 (2021). 34020297 10.1016/j.jhevol.2021.103000

[20] R. Pickering , Australopithecus sediba at 1.977 Ma and implications for the origins of the genus Homo. Science 333, 1421–1423 (2011). 21903808 10.1126/science.1203697

[21] D. Stratford, Sterkfontein Member 4 data. WIReDSpace. https://www.wiredspace.wits.ac.za/handle/10539/32863. Deposited 12 May 2022.

[22] L. Bruxelles , A multiscale stratigraphic investigation of the context of StW 573 ‘Little Foot’ and Member 2, Sterkfontein Caves, South Africa. J. Hum. Evol. 133, 78–98 (2019). 31358185 10.1016/j.jhevol.2019.05.008

[23] L. Bruxelles, R. J. Clarke, R. Maire, R. Ortega, D. Stratford, Stratigraphic analysis of the Sterkfontein StW 573 Australopithecus skeleton and implications for its age. J. Hum. Evol. 70, 36–48 (2014). 24698198 10.1016/j.jhevol.2014.02.014

[24] M. A. Melton, Debris-covered hillslopes of the southern Arizona desert: Consideration of their stability and sediment contribution. J. Geol. 73, 715–729 (1965).

[25] D. Sanders, M. Ostermann, J. Kramers, Quaternary carbonate-rocky talus slope successions (Eastern Alps, Austria): Sedimentary facies and facies architecture. Facies 55, 345–373 (2009).

[26] I. Statham, A scree slope rockfall model. Earth Surf. Processes 1, 43–62 (1976).

[27] F. L. Pérez, Talus fabric and particle morphology on Lassen Peak, California. Geogr. Ann., Ser. A 71, 43–57 (1989).

[28] H. B. S. Cooke, The Sterkfontein bone breccia: A geological note. S. Afr. J. Sci. 35, 204–208 (1938).

[29] J. T. Robinson, Sterkfontein stratigraphy and the significance of the extension site. S. Afr. Archaeol. Bull. 66, 87–107 (1962).

[30] D. N. Stiles, T. C. Partridge, Results of recent archaeological and palaeoenvironmental studies at the Sterkfontein extension site. S. Afr. J. Sci. 75, 346–352 (1979).

[31] M. J. Wilkinson, Geomorphic perspectives on the Sterkfontein australopithecine breccias. J. Archaeol. Sci. 10, 515–529 (1983).

[32] M. Bamford, Pliocene fossil woods from an early hominid cave deposit, Sterkfontein, South Africa. S. Afr. J. Sci. 95, 231–237 (1999).

[33] M. Horn, “Clarifying the stratigraphic boundary between Member 4 and Member 5 of the Sterkfontein Caves, South Africa: A three-dimensional spatial analysis of hominin fossils and stone tools,” MS thesis, University of the Witwatersrand, Johannesburg, South Africa (2021).

[34] S. H. Haughton, Notes on the australopithecine-bearing rocks of the Union of South Africa. S. Afr. J. Geol. 50, 55–59 (1947).

[35] T. C. Partridge, I. B. Watt, The stratigraphy of the Sterkfontein hominid deposit and its relationship to the underground cave system. Palaeontologica Africana 28, 35–40 (1991).

[36] G. Balco, Production rate calculations for cosmic-ray-muon-produced ^10^Be and ^26^Al benchmarked against geological calibration data. Quat. Geochronol. 39, 150–173 (2017).

[37] J. Moggi-Cecchi, F. E. Grine, P. V. Tobias, Early hominid dental remains from Members 4 and 5 of the Sterkfontein Formation (1966-1996 excavations): Catalogue, individual associations, morphological descriptions and initial metrical analysis. J. Hum. Evol. 50, 239–328 (2006). 16309732 10.1016/j.jhevol.2005.08.012

[38] M. Toussaint, G. A. Macho, P. V. Tobias, T. C. Partridge, A. R. Hughes, The third partial skeleton of a late Pliocene hominin (Stw 431) from Sterkfontein, South Africa. S. Afr. J. Sci. 99, 215–223 (2003).

[39] R. J. Clarke, “*Australopithecus* from Sterkfontein Caves, South Africa” in The Palaeobiology of Australopithecus, K. Reed, J. Fleagle, R. Leakey, Eds. (Springer, 2013), pp. 105–123.

[40] K. Nishiizumi , Absolute calibration of ^10^Be AMS standards. Nucl. Instrum. Methods Phys. Res. B 258, 403–413 (2007).

[41] Z. Zhao , A test of the isochron burial dating method on fluvial gravels within the Pulu volcanic sequence, West Kunlun Mountains, China. Quat. Geochronol. 34, 75–80 (2016).

[42] H. Tu, G. Shen, D. Granger, X. Yang, Z. Lai, Isochron ^26^Al/^10^Be burial dating of the Lantian hominin site at Gongwangling in northwestern China. Quat. Geochronol. 41, 174–179 (2017).

[43] K. Kuman, The archaeology of Sterkfontein—past and present. J. Hum. Evol. 27, 471–495 (1994).

[44] C. K. Brain, The Hunters or the Hunted? An Introduction to African Cave Taphonomy (University of Chicago Press, 1981).

[45] J. Kibii, “Comparative taxonomic, taphonomic, and palaeoenvironmental analysis of 4-2.3 million year old australopithecine Cave Infills at Sterkfontein,” PhD thesis, University of the Witwatersrand, Johannesburg, South Africa (2004).

[46] H.B.S. Cooke, Undescribed suid remains from Bolt’s Farm and other Transvaal cave deposits. Palaeontol*. A*fr., 30, 7–23 (1993).

[47] T. D. White, F. C. Howell, H. Gilbert, The earliest Metridiochoerus (Artiodactyla: Suidae) from the Usno Formation, Ethiopia. Trans. R. Soc. S. Afr. 61, 75–79 (2006).

[48] N. T. Boaz, F. C. Howell, M. L. McCrossin, Faunal age of the Usno, Shungura B and Hadar formations, Ethiopia. Nature 300, 633–635 (1982).

[49] J. A. Lee-Thorp, M. Sponheimer, J. Luyt, Tracking changing environments using stable carbon isotopes in fossil tooth enamel: An example from the South African hominin sites. J. Hum. Evol. 53, 595–601 (2007). 17920103 10.1016/j.jhevol.2006.11.020

[50] S. C. Reynolds and J. M. Kibii, Sterkfontein at 75: Review of palaeoenvironments, fauna, and archaeology from the hominin site of Sterkfontein (Gauteng Province, South Africa). Palaeontol*. A*fr. 46, 59–88 (2011).

[51] A. Koutsodendris , (c. 0-4 Ma) cyclostratigraphy for IODP Site U1478 (Mozambique Channel, SW Indian Ocean): Exploring an offshore record of paleoclimate and ecosystem variability in SE Africa. Newsl. Stratigr. 54, 159–181 (2021).

[52] Y. Haile-Selassie , New species from Ethiopia further expands Middle Pliocene hominin diversity. Nature 521, 483–488 (2015). 26017448 10.1038/nature14448

[53] Y. Haile-Selassie , A new hominin foot from Ethiopia shows multiple Pliocene bipedal adaptations. Nature 483, 565–569 (2012). 22460901 10.1038/nature10922

[54] A.-E. Lebatard , Cosmogenic nuclide dating of Sahelanthropus tchadensis and Australopithecus bahrelghazali: Mio-Pliocene hominids from Chad. Proc. Natl. Acad. Sci. U.S.A. 105, 3226–3231 (2008). 18305174 10.1073/pnas.0708015105 PMC2265126

[55] M. G. Leakey , New hominin genus from eastern Africa shows diverse middle Pliocene lineages. Nature 410, 433–440 (2001). 11260704 10.1038/35068500

[56] K. Nishiizumi, Preparation of ^26^Al AMS standards. Nucl. Instrum. Methods Phys. Res. B 223, 388–392 (2004).

[57] J. Chmeleff, F. von Blanckenburg, K. Kossert, D. Jakob, Determination of the ^10^Be half-life by multicollector ICP-MS and liquid scintillation counting. Nucl. Instrum. Methods Phys. Res. B 268, 192–199 (2010).

[58] G. Korschinek , A new value for the half-life of ^10^Be by Heavy-Ion Elastic Recoil Detection and liquid scintillation counting. Nucl. Instrum. Methods Phys. Res. B 268, 187–191 (2010).

[59] G. Balco, C. W. Rovey, An isochron method for cosmogenic-nuclide dating of buried soils and sediments. Am. J. Sci. 308, 1083–1114 (2008).

[60] E. D. Erlanger, D. E. Granger, R. J. Gibbon, Rock uplift rates in South Africa from isochron burial dating of fluvial and marine terraces. Geology 40, 1019–1022 (2012).

[61] P. V. Tobias, A. R. Hughes, The new Witwatersrand University excavation at Sterkfontein: Progress report, some problems, and first results. S. Afr. Archaeol. Bull. 24, 158–169 (1969).

[62] D. Stratford, S. Merlo, S. Brown, The development of a new geospatial framework for the palaeoanthropological site of Sterkfontein Caves, Cradle of Humankind, Gauteng, South Africa. J. Field Archaeol. 41, 211–221 (2016).

[63] N. Lifton, T. Sato, T. J. Dunai, Scaling in situ cosmogenic nuclide production rates using analytical approximations to atmospheric cosmic-ray fluxes. Earth Planet. Sci. Lett. 386, 149–160 (2014).

