# Supplementary material for: Cosmogenic nuclide dating of Australopithecus at Sterkfontein, South Africa
Source: Proc Natl Acad Sci U S A. 2022 Jun 27;119(27):e2123516119. doi: 10.1073/pnas.2123516119 (PMC9271183; doi:10.1073/pnas.2123516119)
Supplement: Supplementary File [file pnas.2123516119.sapp.pdf]

PNAS
www.pnas.org

Supplementary Information for
Cosmogenic nuclide dating of *Australopithecus* at Sterkfontein, South Africa

Darryl E. Granger[1]*, Dominic Stratford[2], Laurent Bruxelles[2,3], Ryan J. Gibbon[4], Ronald J. Clarke[5], Kathleen Kuman[2]
[1]Department of Earth, Atmospheric, and Planetary Sciences, Purdue University; West Lafayette, Indiana, 47907.
[2]School of Geography, Archaeology and Environmental Studies, University of the Witwatersrand; Johannesburg, WITS 2050, South Africa.
[3]Travaux et Recherches Archéologiques sur les Cultures, les Espaces et les Sociétés (TRACES), UMR 5608 of the CNRS, Jean Jaurès University, Toulouse, France, 31058
[4]Cape Town, South Africa
[5]Evolutionary Studies Institute, University of the Witwatersrand; Johannesburg, WITS 2050, South Africa

*corresponding author Darryl Granger.
Email: dgranger@purdue.edu

This PDF file includes:

Supplementary text
Figures S1 to S8
Tables S1 to S2
SI References

Supplementary Text

Measurement of talus cone fabric.

In a talus cone, flattened clasts typically align their lower faces parallel to bedding as they glide down the surface (1). To more accurately quantify the dip of the talus in the surface excavations, clast orientations were measured from exposures of cemented breccia in the deep excavation walls of Member 4. Because the clasts were cemented in place, it was not possible to measure a true dip in three dimensions. Instead, the apparent dip in the plane of the exposure was measured, which is necessarily less than the true dip. The orthogonal orientation of the three main exposed walls of the Member 4 excavation allows a 3-dimensional reconstruction of talus slope direction to be assessed.

Apparent dip angles were calculated by surveying with a total station the ends of the underside surface of all visible flat-bottomed clasts exposed in the profiles that had an elongation rate of greater than 1.6:1. Data were collected from three different walls in the exposed sections: the northern wall (facing south), the eastern wall (facing west), and the southern wall (facing north). Dip angle for each clast was then calculated from the total station data following ref (2). Representative apparent dip measurements and radial histograms of all measurements are shown in Fig. S1.

Member 4 exposed in the basal part of the northern wall, which bears ~115°, shows an apparent dip of 29° ± 17° (mean ± standard deviation) to the east, based on 183 clast measurements (3). In the eastern wall, which bears ~10°, the apparent dip is 27° ± 17° to the north based on 388 measurements (3). In the southern wall, which bears ~105°, the apparent dip is 29° ± 17° to the east, based on 500 measurements (3). Recognizing that that the dip is roughly constant on each wall, the true dip of the talus cone surface where the walls intersect can be estimated trigonometrically. In the northeastern part of the excavation, nearest BH4, the estimated true dip is 42° ± 16° with a dip direction of 62° ± 28°. In the southeastern part of the excavation the estimated true dip is 42° ± 15° with a dip direction of 65° ± 24°. These inferred true dips are near the expected angle of repose for an accumulating talus cone (1,4-6), and indicate talus emanating from the southwest (Fig. S1). In contrast, the predicted dip of the talus based on reconstructions from borehole correlations (7,8) is at a low angle down to the southwest, diametrically opposed to and incompatible with our observations.

Purported Member 4 Equids

In the main text we show evidence that equids assigned to Member 4 can be either improperly assigned during early excavations or intrusive from Member 5 in solution pockets. Here we note that both *Equus* and *Hipparion lybicum* (now genus *Eurygnathohippus*) are listed in a review among Member 4 fauna by Reynolds and Kibii (9). As *Hipparion* was not previously recorded for Member 4, inquiries were made with both authors. Kibii confirmed that the only equid specimens assigned by him to Member 4 (10) are the two postcranial bones discussed in the main text, while Reynolds could only recall that these taxa appear to have been included based on an email from equid specialist V. Eisenmann to P.V. Tobias. However, a January 1997 email from Eisenmann to Tobias with equid identifications from several infills was given to KK with a note asking for help with the provenances, as no catalog numbers were provided. In that letter, only one ‘Member 4 area’ tooth was identified as *Equus*, but it is specimen S94-323, which is now confirmed as found

in decalcified breccia (Fig. S6). The other equid specimens in the letter were all from younger deposits, and *Hipparion* was only noted in the youngest infill.

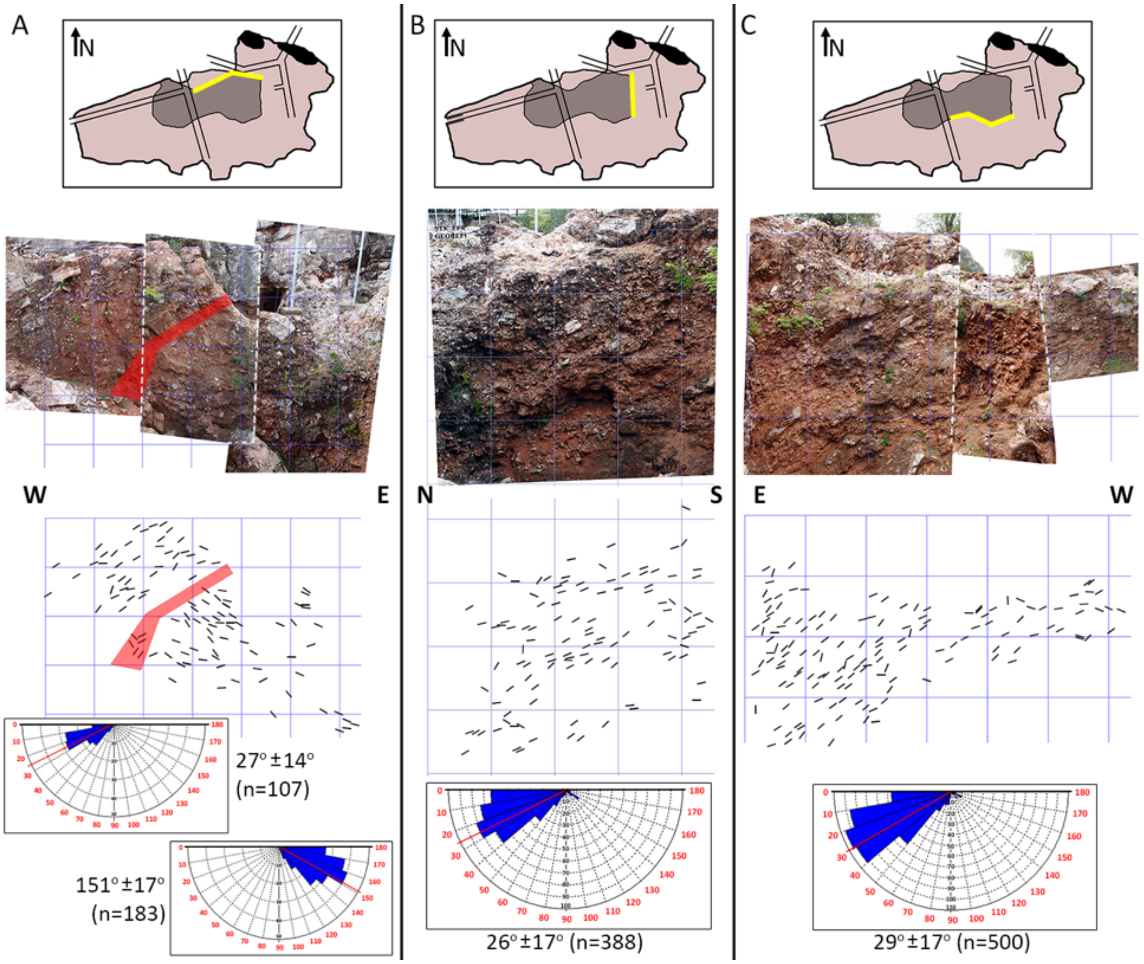

Fig. S1. Measured apparent dips.

Orthorectified photo mosaics of the Member 4 excavation walls, with representative apparent dip measurements shown as oriented lines. Radial plots show all dip measurements with their mean and standard deviation noted (3). Section locations shown in maps (top). (A) North wall, with west-dipping Member 5 overlying east-dipping Member 4, and a transitional zone in red. Radial plots divided into west-dipping and east-dipping clasts, representing Member 5 and Member 4 respectively. (B) East wall, Member 4. (C) South wall, Member 4.

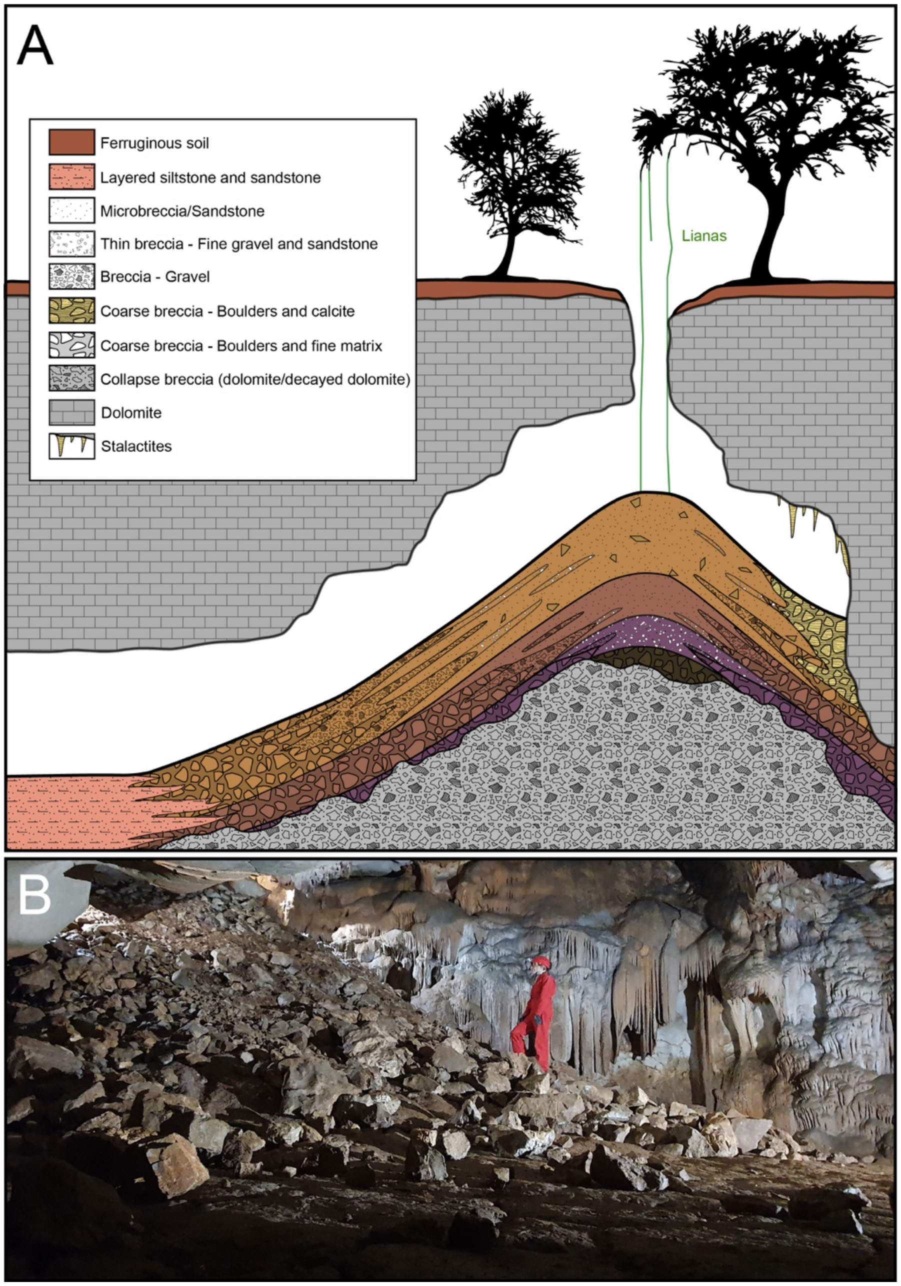

Fig. S2. Typical cave talus cone stratigraphy. (A) Schematic cross section showing that a finer-grained matrix-supported facies accumulates beneath the entrance, grading to a more bouldery, clast-supported facies on the distal flanks. Vegetation, such as liana at Sterkfontein, accumulates near the entrance. Crude bedding is preserved in the proximal and medial parts. Note that colors in the talus cone refer to sequential phases of deposition, while the pattern indicates grain size and texture as denoted in the key. The distal boulder facies may include finer matrix or may be matrix-free and later cemented by calcite derived from dripwater from the cave roof, as at Sterkfontein (Main text, Fig. 2). (B) Typical cave talus illustrating the facies described in (A). Photo from Roquette Cave, Conqueyrac, France, by Carole Bruxelles.

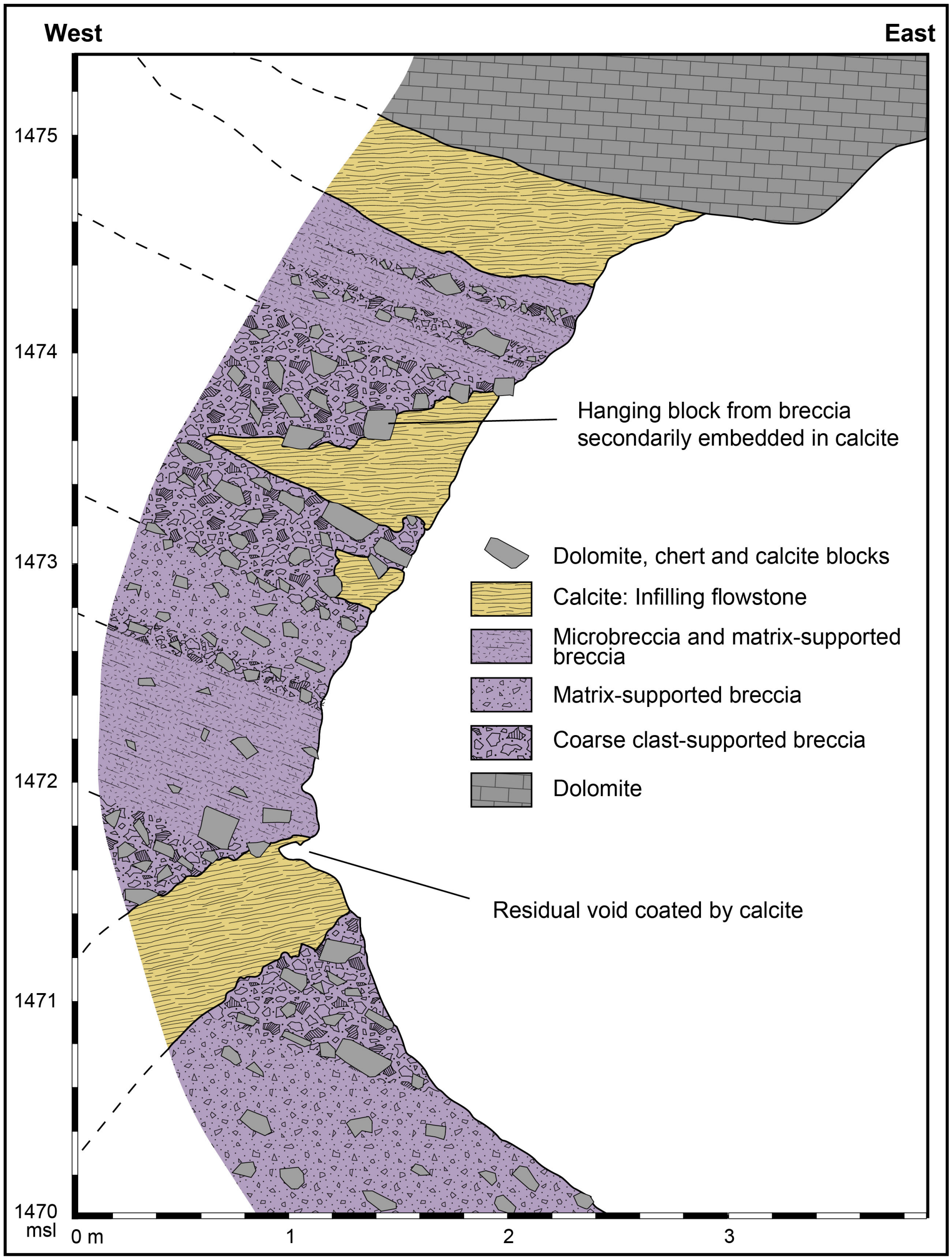

Fig. S3. Stratigraphic section at western end of Fossil Cavern.

Flowstones in Member 4 here are intrusive, filling voids within the breccia, as evidenced by unconformable contacts and breccia embedded in the calcite. One of the best demonstrations is that blocks hanging from the overlying breccia are embedded in the flowstone. This implies that they were already there while the flowstone was developing, filling secondary cavities within M4 breccia. These are typical flowstones in Member 4, and are probably representative of flowstones found in the boreholes (7,8).

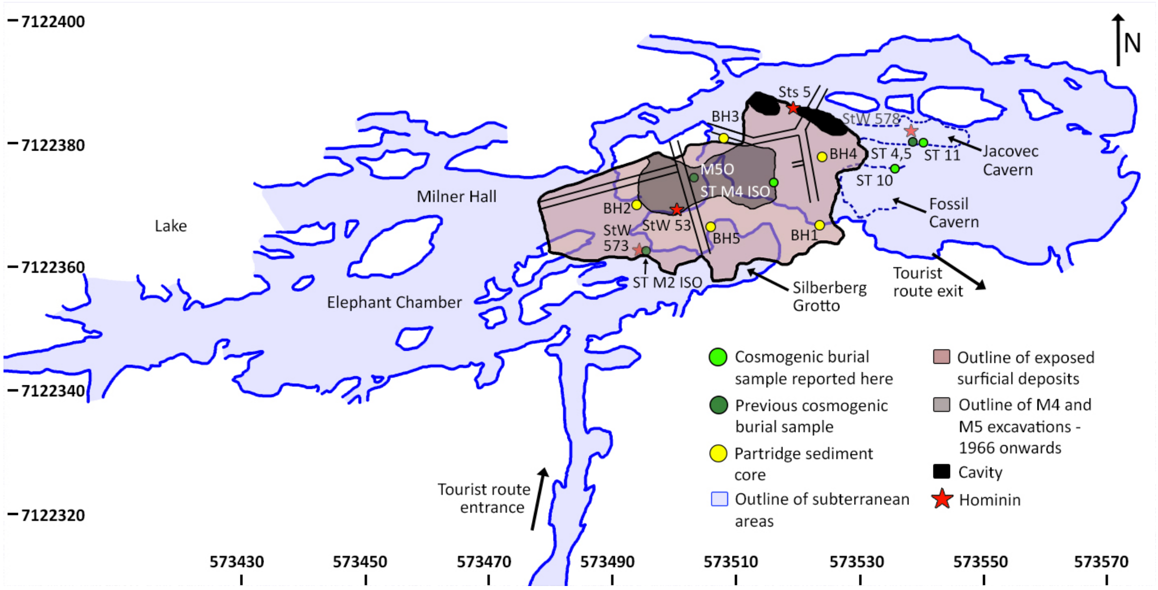

Fig. S4.

Map of Sterkfontein Cave and the surface excavations in the vicinity of the fossil-bearing breccias based on hand-held LiDAR and total station surveys. The locations of boreholes used in refs. (7,8), and selected hominin fossils cited in text are indicated. UTM coordinates following ref. (11). Cosmogenic samples reported here are denoted with light green circles; previously dated cosmogenic samples (12,13) are shown as dark green circles.

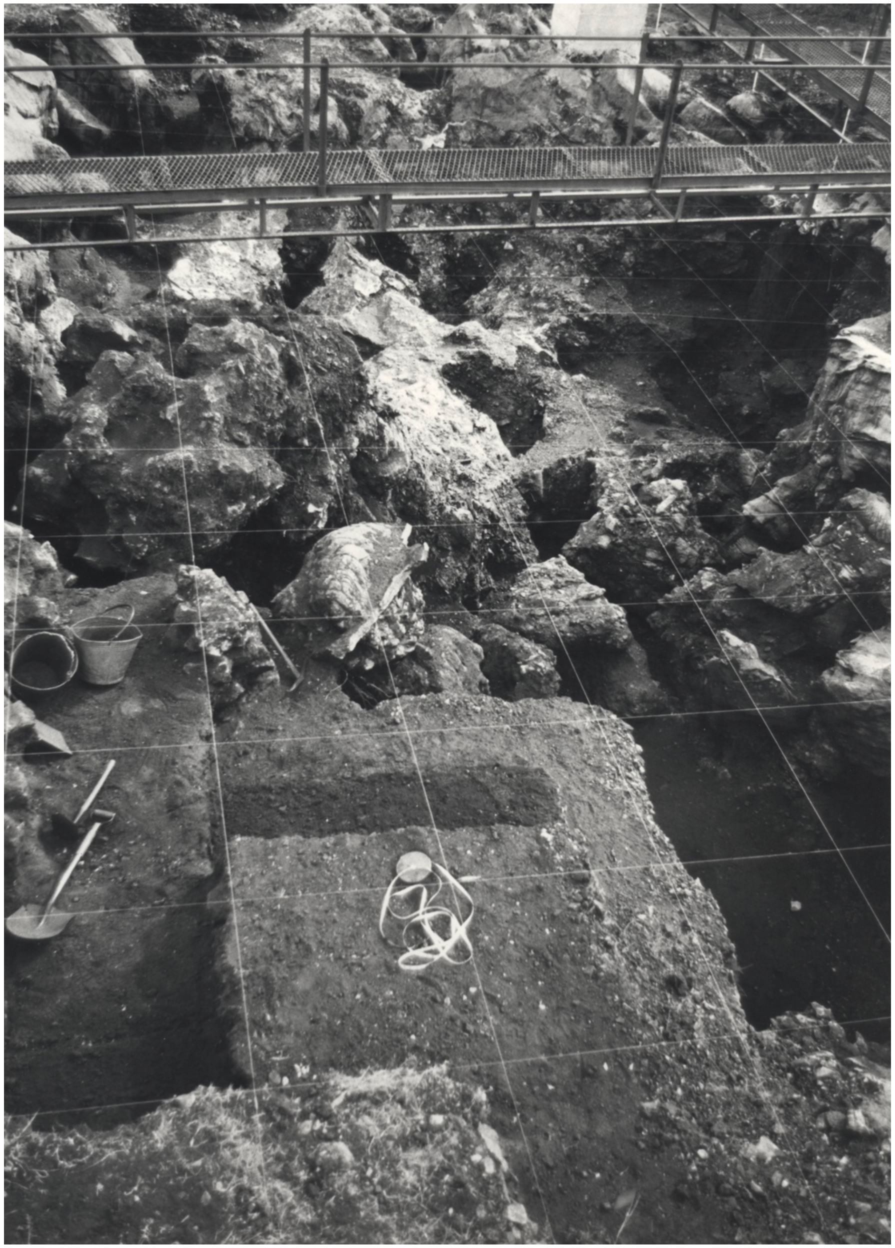

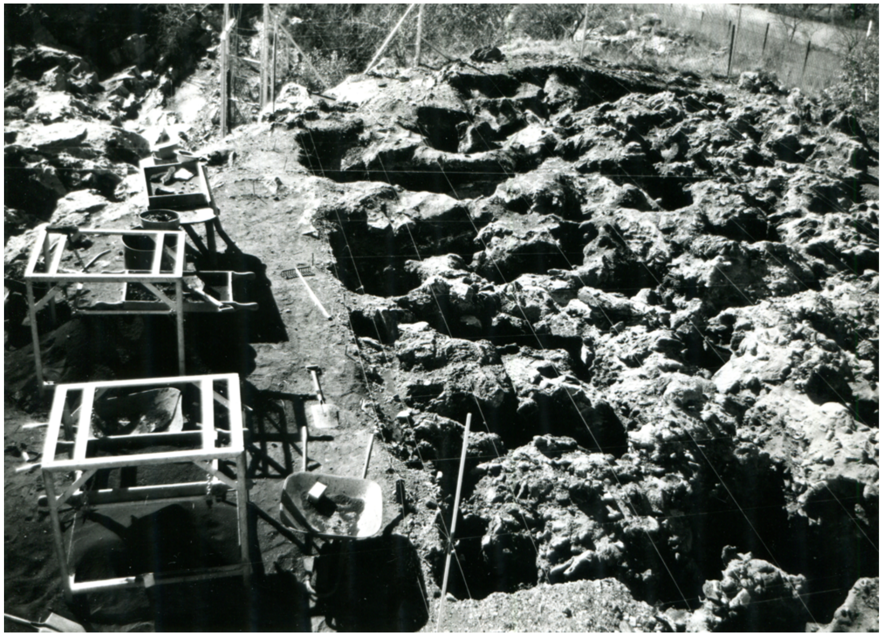

Fig. S5

Historic photos of the surface of Member 4 during excavation of the decalcified breccia in solution pockets (photos by Alun Hughes). Hughes excavated at Sterkfontein beginning by removing looser sediment from solution pockets to expose the intact cemented breccia. Solution pockets were extensive, and the records of their locations made at the time was only photographic. Although this issue was not addressed as a notable source of mixing until the 1990s (14), Hughes in fact often cataloged hominid finds from this eastern part of the site as ‘Member 4 or 5’. Strings mark 3-foot (0.91-m) squares.

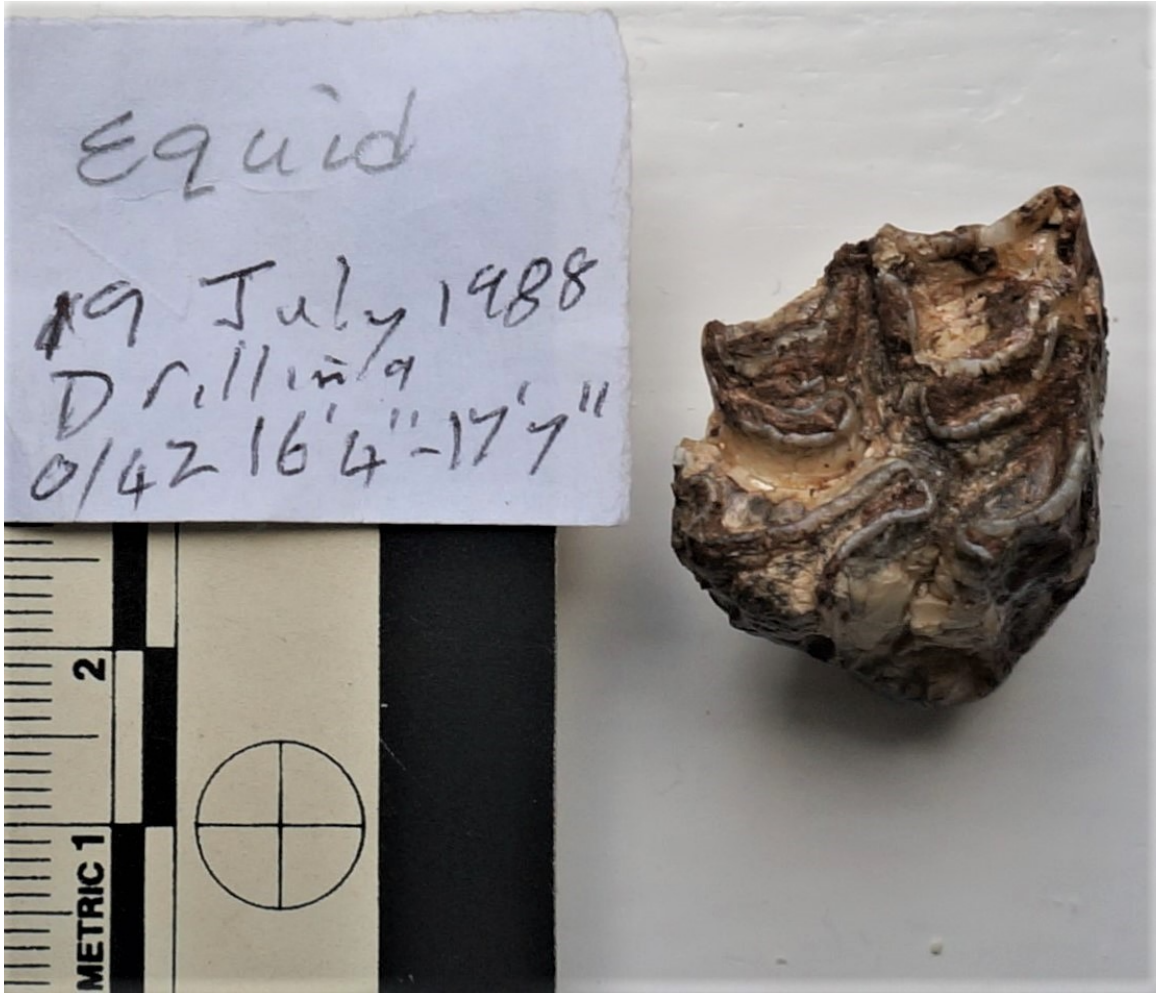

Fig. S6

Equus tooth S94-323 from Square O/42 at 16’4”-17’4” (4.98-5.28 m) below datum, together with its original label. Note the dark staining by manganese indicating its derivation from a solution pocket. Solution pockets typically contain breccia that is darker in color than surrounding calcified breccia, because humic acids dissolve calcium carbonate and mobilize manganese from the dolomite as it breaks down. In such a context, decalcified breccia can admit younger, overlying material (15-17), which filters down to lower levels through disturbance by tree roots and localized subsidence of sediment as breccia loses the support of calcium carbonate. This *Equus* tooth was excavated on 19 July 1988 from the above spit, but RJC also noted on the label a more specific depth of 16’4”-17’7” (4.98-5.36 m) for the breccia block as it was removed for processing.

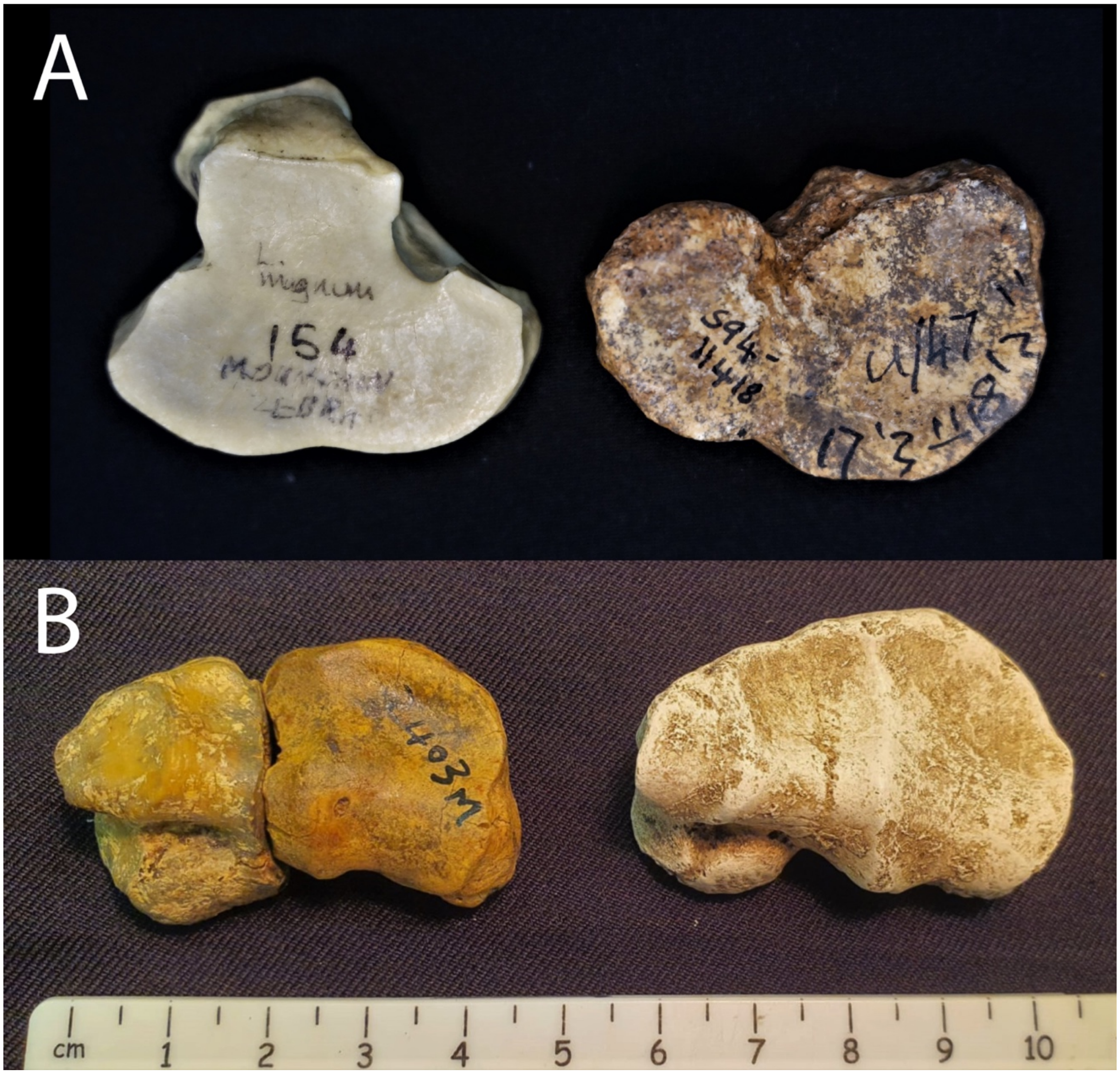

Fig. S7.

Fossil S94-11418, previously identified as an equid magnum, but here identified as a fused unciform and magnum of a Size 3 bovid. (A) Inferior view of the original Sterkfontein fossil (right) compared to a modern equid magnum (left), from a mountain zebra (*Equus zebra zebra*). Note that they are totally different in morphology, whether considering the fused fossil bones or the magnum on its own. (B) Superior view of a cast of S94-11418 (right) compared to the unciform and magnum of a Size 3 bovid from Makapansgat Member 3, taxon unknown. Photos: Recognise Sambo.

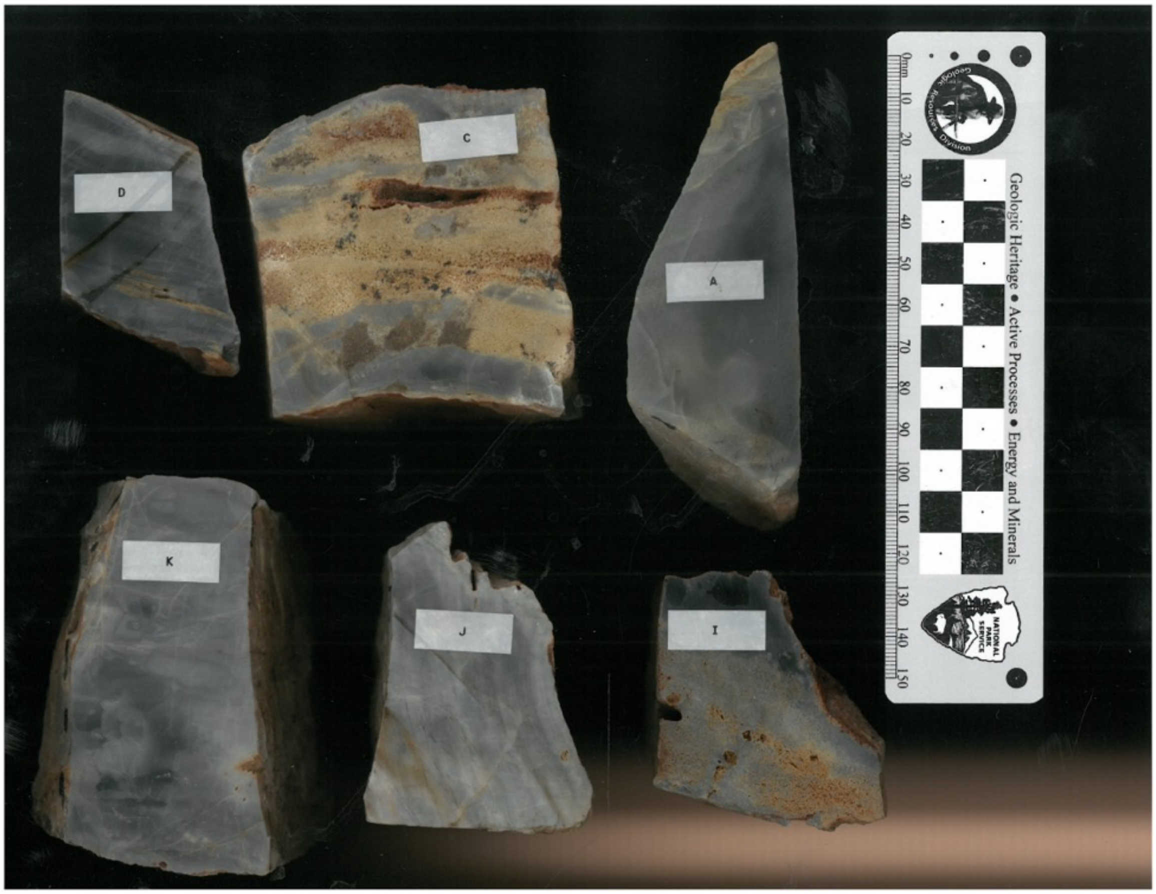

Fig. S8.

Scanned sawn sections of chert blocks analyzed in this study. All blocks show evidence of weathering at the surface prior to deposition in the cave.

Table S1. Cosmogenic nuclide data

Sample	PRIME ID	mass (g)	Be (μg)	[Al] (μg/g)	^{10}Be/^{9}Be (x 10^{-15})	^{26}Al/^{27}Al (x 10^{-15})	[^{10}Be] (10^3 at g^{-1})	[^{26}Al] (10^3 at g^{-1})
ST M4 A	201502710	60.302	290.0	75.0 ± 1.5	297 ± 10	143 ± 7	92.1 ± 3.5	239.9 ± 12.3
ST M4 C	201502711	86.952	279.7	61.6 ± 1.2	694 ± 20	225 ± 35	146.9 ± 4.3	310.1 ± 47.8
ST M4 D	201502712	44.207	288.8	53.7 ± 1.1	744 ± 17	447 ± 14	320.2 ± 7.8	535.3 ± 17.3
ST M4 I	201502713	38.937	285.5	50.4 ± 1.0	853 ± 29	508 ± 17	412.7 ± 14.2	570.7 ± 18.6
ST M4 J	201502714	31.659	283.6	60.6 ± 1.2	619 ± 18	424 ± 14	363.9 ± 10.9	572.9 ± 19.2
ST M4 K	201502715	52.813	278.5	77.7 ± 1.5	1286 ± 27	383 ± 12	449.2 ± 9.6	664.5 ± 20.2
Blank	201502716				11 ± 5	1.2 ± 0.9		
ST 10*	200601222	44.738	337.6	245 ± 12	1234 ± 28	---	627.2 ± 14.2	
ST 10 (2014)*	201402169					146 ± 10		798.3 ± 67.7
ST 11*	200601212	48.426	337.7	215 ± 11	1090 ± 41	106 ± 23	507.9 ± 18.9	
ST 11 (2014)*	201402170					116 ± 6		554.3 ± 43.2
Blank	200600202				8 ± 2	n.m.		
ST 4*	200001797	62.565	562.7	408 ± 20	926 ± 27	60 ± 7	552.2 ± 16.4	
ST 4 (2014)*	201402179					55.7 ± 5.2		507.2 ± 53.7
ST 5*	200001798	23.634	665.0	271 ± 14	227 ± 12	83.5 ± 8.4	411.8 ± 24.4	
ST 5 (2014)*	201402180					87.1 ± 5.2		526.5 ± 41.0
Blank					0 ± 5	n.m.		

*Reported ^{10}Be/^{9}Be is adjusted to the standard of ref. (18). ^{26}Al/^{27}Al re-measured in 2014; reported [^{26}Al] is the average of the two measurements, except ST 10 which is only the 2014 measurement. All other ^{10}Be samples measured at PRIME Lab against standards reported in refs (18). All ^{26}Al measured at PRIME Lab against standards reported in (19).

Table S2. Values used for age calculations

Sample	depth (m)	density (g cm^{-3})	$P_{10,pb}^{*}$ (at g^{-1} yr^{-1})	$P_{26,pb}^{*}$	P_{10} (at g^{-1} yr^{-1})	P_{26}/P_{10}	Calculated Age (My)
ST M4 A	10	2.0	0.0295	0.2503	9.8 ± 1.6	6.8	3.41 ± 0.11
ST M4 C	10	2.0	0.0295	0.2503	9.8 ± 1.6	6.8	(isochron)
ST M4 D	10	2.0	0.0295	0.2503	9.8 ± 1.6	6.8	
ST M4 I	10	2.0	0.0295	0.2503	9.8 ± 1.6	6.8	
ST M4 J	10	2.0	0.0295	0.2503	9.8 ± 1.6	6.8	
ST M4 K	10	2.0	0.0295	0.2503	9.8 ± 1.6	6.8	
ST 10	11	2.8	0.0186	0.1511	9.8 ± 1.6	6.8	3.49 ± 0.19
ST 11	29	2.8	0.0059	0.0437	9.8 ± 1.6	6.8	3.63 ± 0.13
ST 4	29	2.8	0.0059	0.0437	9.8 ± 1.6	6.8	3.95 ± 0.21
ST 5	29	2.8	0.0059	0.0437	9.8 ± 1.6	6.8	3.39 ± 0.16

*calculated using ref (20)

SI References

1. F. L. Pérez., Talus fabric and particle morphology on Lassen Peak, California. *Geografiska Annaler: Series A, Physical Geography*, **71**, 43-57 (1989). doi:10.1080/04353676.1989.11880272
2. S. J. P. McPherron, Artifact orientations and site formation processes from total station proveniences. *Journal of Archaeological Science* **32**, 1003-1014 (2005). doi:10.1016/j.jas.2005.01.015
3. Stratford, D., Sterkfontein Member 4 data (2022). https://www.wiredspace.wits.ac.za/handle/10539/32863. Deposited 12 May 2022.
4. M. A. Melton, Debris-covered hillslopes of the southern Arizona desert: consideration of their stability and sediment contribution. *The Journal of Geology,* **73**, 715-729 (1965). doi:10.1086/627112
5. D. Sanders, M. Ostermann, J. Kramers, Quaternary carbonate-rocky talus slope successions (Eastern Alps, Austria): sedimentary facies and facies architecture. *Facies* **55**, 345-373 (2009). doi:10.1007/s10347-008-0175-z
6. I. Statham, A scree slope rockfall model. *Earth Surface Processes* **1**, 43-62 (1976). doi:10.1002/esp.3290010106
7. R. Pickering, J. D. Kramers, Re-appraisal of the stratigraphy and determination of new U-Pb dates for the Sterkfontein hominin site, South Africa. *Journal of Human Evolution* **59**, 70-86 (2010). doi:10.1016/j.hevol.2010.03.014
8. D. N. Stiles, T. C. Partridge, Results of recent archaeological and palaeoenvironmental studies at the Sterkfontein extension site. *South African Journal of Science* **75**, 346-352 (1979).
9. S. C. Reynolds and J. M. Kibii, Sterkfontein at 75: review of palaeoenvironments, fauna, and archaeology from the hominin site of Sterkfontein (Gauteng Province, South Africa). *Palaeontologia africana*, **46**, 59-88 (2011).
10. J. Kibii, “Comparative Taxonomic, Taphonomic, and Palaeoenvironmental Analysis of 4-2.3 Million Year Old Australopithecine Cave Infills at Sterkfontein” PhD thesis, University of the Witwatersrand, Johannesburg, South Africa (2004).
11. D. Stratford, S. Merlo, S. Brown, The development of a new geospatial framework for the palaeoanthropological site of Sterkfontein Caves, Cradle of Humankind, Gauteng, South Africa. *Journal of Field Archaeology* **41**, 211-221 (2016). doi:10.1080/00934690.2016.1157679

12. T. C. Partridge, D. E. Granger, M. W. Caffee, R. J. Clarke, Lower Pliocene hominid remains from Sterkfontein. *Science* **300**, 607-612 (2003). doi:10.1126/science.1081651
13. D. E. Granger, R. J. Gibbon, K. Kuman, R. J. Clarke, L. Bruxelles, M. W. Caffee, New cosmogenic burial ages for Sterkfontein member 2 Australopithecus and member 5 Oldowan. *Nature* **522**, 85-88 (2015). doi:10.1038/nature14268
14. Clarke 1994
15. K. Kuman, R. J. Clarke, Stratigraphy, artefact industries and hominid associations for Sterkfontein, Member 5. *Journal of Human Evolution* **38**, 827-847 (2000).
16. D. N. Stiles, T. C. Partridge, Results of recent archaeological and palaeoenvironmental studies at the Sterkfontein extension site. *South African Journal of Science* **75**, 346-352 (1979).
17. K. Kuman, The archaeology of Sterkfontein—past and present. *Journal of Human Evolution* **27** 471-495 (1994).
18. K. Nishiizumi, M. Imamura, M. W. Caffee, J. R. Southon, R. C. Finkel, J. McAninch, Absolute calibration of ^{10}Be AMS standards. *Nuclear Instruments and Methods in Physics Research Section B: Beam Interactions with Materials and Atoms*, **258**, 403-413 (2007). doi:10.1016/j.nimb.2007.01.297
19. K. Nishiizumi, Preparation of ^{26}Al AMS standards. *Nuclear Instruments and Methods in Physics Research Section B: Beam Interactions with Materials and Atoms* **223**, 388-392 (2004). doi: 10.1016/j.nimb.2004.04.075
20. G. Balco, Production rate calculations for cosmic-ray-muon-produced ^{10}Be and ^{26}Al benchmarked against geological calibration data, *Quaternary Geochronology* **39**, 150-173 (2017). doi: 10.1016/j.quageo.2017.02.001
